# Distributed medical education in Canada

**Published:** 2018-03-27

**Authors:** Rachel Ellaway, Joanna Bates

**Affiliations:** 1 Department of Community Health Sciences, Cumming School of Medicine, University of Calgary, Alberta, Canada; 2 Office of Health and Medical Education Scholarship, Cumming School of Medicine, University of Calgary, Alberta, Canada; 3 Department of Family Practice, Faculty of Medicine, University of British Columbia, British Columbia, Canada

Canada is geographically immense and highly diverse. We are bounded by the Atlantic Ocean in the East, the Pacific Ocean in the West, and the Arctic Ocean in the North, and we have the longest continuous border in the world with our southern neighbour (more than 4,000 km). Canada encompasses arctic, rain forest, boreal forest, mountain, prairie, and desert regions, we have two official languages, ten provinces and three territories, which together span five time zones. We have a population of just over 35 million that includes many Indigenous peoples as well as immigrants and the descendants of immigrants from all around the world. Despite the size of its population, Canada is sparsely populated at four people per square kilometre, compared, say, to the Netherlands (501/km), United Kingdom (271/km), France (123/km), and the United States (33/km).^1,2^ Like Australia, a large proportion of our population lives in a relatively small number of cities in the south of the country.

Nevertheless, Canada has had a long history of medical education situated primarily in urban faculties of medicine and associated tertiary care academic health science centres (AHSCs). The Flexner report of 1910 that marked a deliberate centralization of medical education on universities and teaching hospitals applied to Canada as well as the US^2^ and is one of the reasons for the many similarities between the US and Canadian medical education systems. Despite this long trend towards centralization (and maybe even to some extent in response to it), in recent years we have seen a major diversification of training contexts beyond the AHSC. Indeed, such is the extent of this change that virtually every medical student and every postgraduate trainee in Canada today spends some time outside of their academic centre during their training.^3^ The practice of training outside the AHSC context has been termed ‘distributed medical education’ (DME) and this special edition of the Canadian Medical Education Journal presents a diverse body of research related to DME from across Canada.

## What is Canadian DME?

The development of DME in Canada has not simply been a matter of swapping AHSC training contexts for their non-AHSC counterparts. It has involved a radical diversification of training contexts so that our learners are now training in places that reflect the many locations, cultures, and types of communities that make up the Canadian healthcare landscape. These settings are not just “somewhere else;” each and every one of them presents new and emerging opportunities and challenges, to learners, to their teachers, and to medical education as a whole. It is arguable that this shift to DME reflects a fundamental democratization of medical education, and at least a partial dismantling of the separation between the worlds of academic medicine and those of the populations they ostensibly serve.^4,5^

DME is not only diverse in comparison to AHSC training contexts, it is also diverse in how different schools have approached it. After all, each of Canada’s 17 medical schools, despite being accredited to common standards, is quite different. Some schools are based in very large cities, have large medical student intakes, and continue to train in a relatively small number of large tertiary hospitals. Others are located in much smaller centres, have smaller intakes, and have long made use of non-AHSC training contexts. Indeed, those programs that have been established or expanded within the context of the DME revolution naturally incorporated DME into their structures. The Northern Ontario School of Medicine (NOSM) for example trains its learners in more than 90 discrete communities, and Université de Sherbrooke operates two regional campuses, both far from the main university site.^6^

There have been a number of drivers for distributing medical education, including: teaching hospitals providing diminishing learning opportunities;^7^ the adoption of social accountability mandates to address workforce needs and health inequities;^8^ the expansion of undergraduate and postgraduate training programs beyond the capacity of AHSCs; and political imperatives for medical schools to better meet the needs of underserved populations.^9^ There have also been differing enablers for DME. For instance, provincial funding has been an essential component in many DME initiatives but not all. Similarly, political backing has been an essential part of some DME initiatives but not for others.

The development of lower cost and increasingly robust learning technologies has also enabled and shaped much of Canadian DME. Videoconferencing in particular has allowed some classes and teachers to interact meaningfully and synchronously over great distances; curricula and learning resources have become much more accessible online; and the shift to digital library collections has made the need to be close to a physical library a thing of the past. How different schools have used technology, however, has also differed according to context.

We can therefore say that the expansion of DME in Canada took place in the context of a confluence of need, intent, and emerging means. It seems unlikely that it could have happened any earlier or any later, at least not in the same way.

## Distribution as an organizing principle in Canadian medical education

While the scale of the recent DME expansion is notable, we should acknowledge that distributed medical education is not a recent innovation; many programs have long included some extramural teaching, electives, or placements. For instance, the Northern Ontario Medical Education Corporation (NOMEC) and the Northwestern Ontario Medical Programme (NOMP) pre-existed the NOSM, and the Rural Ontario Medical Program (ROMP) has been arranging core and elective experiences for medical students across the province for more than thirty years.

There are different models of distribution in medical education across Canada. Some programs have gone for variations on the hub and spoke model, involving one or more regional centres that often have smaller sites associated with them (such as at UBC, McMaster, Western, University of Toronto, Sherbrooke, l’Université de Montreal, and Dalhousie). Other schools have retained the academic centre but with multiple smaller associated sites (such as at Ottawa, Alberta, Calgary, Queens, Laval, and Memorial). Others have set up clinical campuses, where students spend their full clerkship year (such as Manitoba, Saskatchewan, and McGill). Many schools are now offering longitudinal integrated clerkships (LICs) in regional and rural sites as an alternative to AHSC-based block rotations.^10^ The NOSM is intrinsically distributed with its two academic centres and its many larger and smaller sites distributed across Northern Ontario, while both Sherbrooke and Dalhousie have set up regional campuses in neighbouring New Brunswick, one of the two provinces in Canada without its own medical school.

Distribution is not just about geography; it also involves negotiating differing levels of autonomy. Some programs devolve much responsibility to their distributed sites; others retain a great deal of central control. Some are very active at engaging their distributed faculty in running the program as a whole, while others manage everything from the centre. The political tensions associated with DME contributed to distributed schools in other countries breaking into separate and distinct schools (such as the Peninsula and Leicester-Warwick schools in the UK). While this has not happened in Canada, it is still a possibility. Nevertheless, attention to diversity and devolution can be seen as a strength rather than a weakness in the Canadian approach. There may also be something in the Canadian psyche that seems to steer us more towards peacekeeping collaborations to the benefit of all parties than towards independent solitudes. This is certainly a topic that needs further exploration and evaluation.

## Directions in DME research

One of the recurring challenges in DME has been demonstrating equivalence of opportunity across different sites, particularly in the context of accreditation standards that require that learners at one site should never be disadvantaged relative to learners at any other site. While this is an important concern, we would argue that this has diverted attention from the value of different medical education experiences at different sites. One direction for future DME research is in exploring the distinctiveness of DME experiences and the ways in which they can more constructively contribute to individual learning paths. We need to understand how learners adapt to new and differing contexts as they move through DME activities, and we need to understand the longer-term impact of these experiences on their flexibility, resilience and developing professional identity.

Our learners are not our only concern. We must also be clearer about how DME programs impact the health care professionals, the healthcare systems, and the communities with which they intersect. We know that some DME activities have local socio-economic impacts^11^ and can transform communities and their inhabitants.^12^ Postgraduate trainees rotating through DME sites can bring new skills, knowledge and capacity to communities and their healthcare teams. We know that even undergraduate learners engaged in some DME activities, in particular LICs, can make a positive contribution to local healthcare but only after learners have spent many weeks in a particular context.^13^ Duration, immersion, and the nature of the collaborations that underpin DME initiatives also deserve more attention, as do the economic impacts of DME in terms of finances, time, and its effect on healthcare service delivery.

It is interesting to see that research on DME in recent years has shifted from justification and descriptive studies to an increasingly historically and culturally situated discourse about the nature of medical education in Canada as a whole. Indeed, we would argue that, despite the ongoing centralization of scholarship in our field on AHSCs, it is DME that is the ground where much of the future of Canadian medical education is being shaped. In large part this is because distribution has been (and continues to be) a “disruptive technology”^14^ in Canadian medical education. The widespread uptake of DME has established a new field of practice and inquiry and it has disrupted many of the traditional practices and cultures from which it has emerged. For instance, if DME learning outcomes turn out to be better, richer, or more socially accountable than those from traditional AHSC streams, then we may see a move to further decentralize medical education. Alternatively, it may be that DME may prove a superior option for some students but not for all,^15^ or we may explore how we can use both AHSCs and DME settings selectively according to their strengths at supporting different kinds of learning at different levels of training to afford different kinds of learning outcomes.

While we have understandably focused on Canadian DME in this journal, we need to acknowledge that DME is a worldwide phenomenon. However, DME is highly context-dependent and to that end, while DME in Canada shares some factors with DME in other contexts (Australia’s size and population density, the United States’ geography and medical education system), contextual factors (such as geography, climate, culture, and history) in Canada makes DME here distinct from approaches in other countries. For instance, DME in the UK involves much smaller distances and much less geographical variability alongside a much more centralized healthcare system (the NHS) compared to Canada. The generalizability of DME research in any context is therefore another key issue that needs further exploration, both in terms of translating DME research into practice, and in terms of the questions and problems that this research needs to address.

## In this edition …

It is in this context that we present this special edition of the Canadian Medical Education Journal. The range of topics, domains of medical education, study contexts, and methodologies employed in these papers reflects the rich diversity of the Canadian DME landscape. We see this variety in the UME context: Brown and authors outline how student-produced medical theatre grounded in local health needs and experiences can promote social accountability and the education of health professionals in the context of regional medical campuses; Lévesque et al. evaluate an evidence-based medicine educational intervention in a regional medical campus that helps medical students learn evidence informed medical practice and its outcomes; and Maar and colleagues describe the Community Engagement through Research (CETR) program at the Northern Ontario School of Medicine (NOSM) and the ways in which students and community members appreciated the application of the research to real community problems. In the PGME context, Jattan et al. surveyed urban and rural family medicine residents at the University of Manitoba and found that there are fewer teaching opportunities for rural family medicine residents compared to urban residents. Interprofessional issues are reflected in the paper by Walmsley et al. looking at the challenges and opportunities in regional medical campuses in providing effective interprofessional education (IPE). Faculty matters are considered by Zelek and Goertzen, who suggest that our present understanding of faculty engagement is limited and insist that we use a more sophisticated understanding of the many extrinsic and intrinsic motivators to better engage faculty involved in distributed medical education. Medical careers are explored by Levesque and colleagues by looking at the factors that influence physicians’ decisions to establish and maintain their practice in a particular region, concluding that a regional medical campus can have strong direct and indirect effects on recruitment and retention decisions. Utzschneider and Landy also consider career issues by showing that individuals completing a medical program in a Francophone regional medical campus in New Brunswick were more likely to practice in the province or in Atlantic Canada generally. At a broader level, Strasser et al. describe how community engagement can contribute to a school’s social accountability mission, while Wooster et al. describe how a locally developed educational organizational structure along with a strong community-focus and suitable patient volumes and complexity can support valuable experiential learning at distributed sites. And finally, Lemky at al. systematically identify and evaluate methods of economic assessment relevant to distributed medical education.

DME research is clearly more than a matter of describing and evaluating new campuses and programs. It is a rich intersectional space where many of the central issues that we face in medical education can be revisited, and new understanding developed, not just for DME but for all of medical education, distributed or otherwise. The situatedness of this research and the insight it can give to social value of different medical education practices is particularly notable. At a time when research and the knowledge it produces is increasingly required to be socially robust,^16^ the DME context and the work coming out of it is a critical component of the Canadian medical education landscape.

## Conclusion

In summary, the DME revolution in Canada has been a particularly Canadian response to the different (and to an extent unmet) healthcare needs of the Canadian people. The significance of research in to DME is not because we have a single Canadian way of doing DME but rather because this research reflects our highly diverse approaches to DME, which, in turn, reflect our diverse populations, geographies, and local approaches to medical education. Canadian DME reflects the heart of Canadian medical education today.
